# Comprehensive Identification and Alternative Splicing of Microexons in *Drosophila*

**DOI:** 10.3389/fgene.2021.642602

**Published:** 2021-03-30

**Authors:** Ting-Lin Pang, Zhan Ding, Shao-Bo Liang, Liang Li, Bei Zhang, Yu Zhang, Yu-Jie Fan, Yong-Zhen Xu

**Affiliations:** ^1^University of Chinese Academy of Sciences, Beijing, China; ^2^Key Laboratory of Insect Developmental and Evolutionary Biology, Center for Excellence in Molecular Plant Sciences, Chinese Academy of Sciences, Shanghai, China; ^3^RNA Institute, State Key Laboratory of Virology, Hubei Key Laboratory of Cell Homeostasis, College of Life Science, Wuhan University, Wuhan, China

**Keywords:** microexon, *Drosophila*, alternative splicing, *ce-Tophat*, sex determination

## Abstract

Interrupted exons in the pre-mRNA transcripts are ligated together through RNA splicing, which plays a critical role in the regulation of gene expression. Exons with a length ≤ 30 nt are defined as microexons that are unique in identification. However, microexons, especially those shorter than 8 nt, have not been well studied in many organisms due to difficulties in mapping short segments from sequencing reads. Here, we analyzed mRNA-seq data from a variety of *Drosophila* samples with a newly developed bioinformatic tool, ce-TopHat. In addition to the Flybase annotated, 465 new microexons were identified. Differentially alternatively spliced (AS) microexons were investigated between the *Drosophila* tissues (head, body, and gonad) and genders. Most of the AS microexons were found in the head and two AS microexons were identified in the sex-determination pathway gene *fruitless*.

## Introduction

Ribonucleic acid splicing removes intronic sequences and ligates exonic sequences in eukaryotic cells. This is catalyzed by the spliceosome, a large and dynamic RNA–protein complex composed of five small nuclear RNAs (U1, U2, U4, U5, and U6 snRNAs) and over 100 proteins (Will and Luhrmann, 2011). On the Ensembl website, 595,500 and 83,529 exons can be retrieved from human and *Drosophila* transcriptomes, respectively. The length of exons varies from two to 11,555 nts (Sakharkar et al., 2004), usually longer in the lower eukaryotes than in the higher eukaryotes (Berget, 1995).

Definition of microexons, the range of microexon length, varies in different reports, such as ≤ 51 nt (Volfovsky et al., 2003; Irimia et al., 2014; Yang and Chen, 2014), 3–27 nt (Li et al., 2015), and 3–30 nt (Ustianenko et al., 2017). Here, we defined exons with length ≤ 30 nt are microexons. It was first reported that two 5-nt exons were found in the *Drosophila Ubx* gene, and a 6-nt exon was in the chicken cardiac troponin T (*TnT*) gene (Beachy et al., 1985; Cooper and Ordahl, 1985); and soon after a 30-nt and a 3-nt exon were identified from the rat and mouse *Ncam* genes, respectively (Small et al., 1988; Santoni et al., 1989). In a mammal, microexons play a crucial role in the development and maintenance of neuronal functions (Irimia et al., 2014; Li et al., 2015), and there are 15,688 microexons in humans according to transcriptome annotation (version: GRCH38.96).

Early annotation tools had a poor ability to find microexons until a systematical alignment between cDNA and genomic DNA sequences identified 319 microexons from four genomes (Volfovsky et al., 2003). In the past decade, many tools have been developed to predict microexons, including the commonly used TopHat (Trapnell et al., 2009) and HISAT (Kim et al., 2015) and customized software GMAP (Wu and Watanabe, 2005), Olego (Wu et al., 2013), VAST-TOOLS (Irimia et al., 2014), and ATMap (Li et al., 2015). However, there are still limitations in the prediction of microexons. First, due to their alignment principles, TopHat and HISAT have difficulty accurately aligning and predicting the 3–7 nt very short microexons. Second, although Olego, VAST-TOOLS, and ATMap can partly predict the 3–7 nt microexons, their principles ignore the ends of sequencing reads that could not be mapped but may contribute to microexons identification. Third, most of these tools were originally developed based on mammalian transcriptomes, which could undervalue microexons in other organisms.

Alternative splicing plays critical roles in the regulation of gen = −0987 \e expression, generating multiple RNA isoforms from one gene and thereby extended proteome in eukaryotes. More than 95% of genes are alternatively spliced (Pan et al., 2008). The first reported alternatively spliced (AS) microexon is a 9-nt exon in the *Drosophila fasciclin I*, resulting in altered binding specificity of Fasciclin I (McAllister et al., 1992). Another interesting early example is a 3-nt microexon in the *Drosophila TnT* gene, which is specifically skipped in the larva’s subcutaneous muscular system, resulting in a protein with a missed lysine residue (Benoist et al., 1998). Later studies in mice have revealed that spatial structures and the domains of proteins were changed due to AS of microexons, such as splicing of microexon 20 in the *ITSN1* gene (Tsyba et al., 2008) and microexon L in the *Protrudin* gene (Ohnishi et al., 2014). AS of microexons could also introduce a phosphorylation site that alters the original protein function. For example, the microexon E8a in the *LSD1* gene encodes a phosphorylated site, Thr369b (Toffolo et al., 2014). On the other hand, retained microexons may introduce a premature stop codon, leading the transcript into an NMD-mediated degradation pathway (Li et al., 2015).

Systematic studies of microexons are mainly related to the mammalian neural systems, and little is known about the development of other organisms. To extensively identify microexons and their alternative splicing, we used in-house RNA deep sequencing data from a variety of *Drosophila* samples and developed an improved computational model ce-TopHat. In total, we identified 985 reliable microexons, of which 465 are novel. Subsequent analyses revealed that the microexons in coding regions are more prone to be alternatively spliced than exons with length > 30 nt. Differentially AS microexons have been investigated between tissues and genders. Over 65% of the AS microexons are found in the head, and two AS microexons in the sex-determination pathway gene *fruitless* are investigated.

## Materials and Methods

### Fly Strain, Culture, and Samples

Stocks and crosses of the wild-type *w*^1118^ isogenic *Drosophila* strain (BDSC 5905) were maintained and cultured on standard cornmeal agar medium (Qiu et al., 2019). Embryos of 18 h wandering third instar larvae and adults of 24 h were collected, respectively. Heads and abdomen-thorax (bodies) of adults were dismembered using liquid nitrogen; the gonads were dissected in ice-cold PBS from fresh flies and collected, respectively, for further RNA extraction.

### RNA-Seq

Ribonucleic acid samples were prepared as described (Li et al., 2020), and the construction of cDNA libraries and sequencing were performed using Illumina Hi-Seq 2000 (Stark et al., 2019). The head, body, and gonads of the female and male *Drosophila* were sequenced in the form of 150 bp fr-firststrand pair-end reads, and the embryo, 3L larvae, and adults were sequenced in the form of 100 bp fr-untstranded pair-end reads. Most of these sequencing data are provided by members of Xu Lab from their unpublished projects.

### Reads Mapping and Remapping

All raw data were trimmed and filtered by FastQC v0.11.5 and cutadapt v1.15, and the clean reads were then mapped to the *Drosophila* genome (dmel_r6.15 version) using TopHat (v2.1.1), in which 2-nt mismatches were allowed with other default parameters (Supplementary Figure 1A).

Unmapped reads were then remapped by an improved computational model, ce-TopHat (cut-end TopHat). First, the ce-TopHat removed 3–7 nt from the two ends of each unmapped read, respectively. These new reads were defined as the cut-end reads, which were remapped to the genome using TopHat (mismatch = 0 and anchor ≥ 8). Second, for the successfully remapped cut-end reads, ce-TopHat will find their genomic locations, which must meet three conditions: (1) mismatch = 0, (2) the cut-end part is flanked by classical splicing site sequences AG/____/GT, and (3) it is located within the gene range and the gap is between 20 and 10,000 nt. The finally met cut-end parts were then recombined to form complete and unique genomic localized reads for the construction of transcripts.

### Identification of Microexons

After mapping and remapping, all junction reads were retrieved from the reconstructed transcriptome and divided into two groups: multi-junction reads and single-junction reads (Supplementary Figure 1B). These junction reads, if containing a ≤30 nt junction part, would have a potential sequence of microexon. If the middle junction part in a multi-junction read is ≤30 nt and not annotated as an exon, it will be defined as a novel microexon. For the two ends of the single-junction reads, if it is ≤30 nt but cannot be correctly mapped to an annotated exon, a further extension is performed by other sequencing reads.

### Determination of Reliable Microexons

The microexons predicted by TopHat or ce-TopHat were defined as microexon candidates. To improve the reliability, the following screening processes were used (Supplementary Figure 1C): (1) unique exon junction reads ≥2; (2) total exon junction reads ≥3; (3) at least one multi-junction reads support; and (4) the length of both sides of the novel multi-junction reads must be ≥8 nt. If the candidates of 8–30 nt in length satisfied the first two conditions or the candidates of 3–7 nt in length satisfied all the four conditions, they were defined as reliable microexons.

### RT-PCR

Total RNAs from *Drosophila* samples were isolated by TRIzol (Ambion) and treated with RNase-free DNase I (Invitrogen). For RT-PCR, cDNAs were reverse transcribed using RevertAid Reverse Transcriptase (Thermo) and amplified by Ex-Taq (TaKaRa). All used primers are listed in Supplementary Table 1.

### Alternative Splicing Analysis

Alternative splicing events were determined by the reconstructed *Drosophila* transcriptome as described above. The PSI and Differential PSI (ΔPSI) of each alternative splicing event were calculated by the Perl scripts, in which events with a difference >0.05 were considered as significantly different between samples (Shen et al., 2014; Hartley and Mullikin, 2016; Wu et al., 2018). In the “exon-microexon-exon” model, microexons with 1 > PSI > 0 were defined as alternatively spliced (AS) microexons, and microexons with PSI = 1 were defined as constitutively spiced (CS) microexons. For comparison, longer exons (> 30 nt) were retrieved and analyzed using the same procedure.

### Splice Sites and GO Analyses

Consensus and conservation of splice sites were analyzed by Weblogo as described (Crooks et al., 2004). Briefly, the 5′SS and 3′SS were extracted by Perl, in which the 5′SS sequences contained the last 3 nt in the upstream exon and the first 6 nt in the intron, and the 3′SS sequences contained the last 20 nt in the intron and the first 3 nt in the downstream exon. Those sequences were also analyzed for the strength of splicing sites (binding ability to the spliceosome) by MaxEntScan online software as described (Yeo and Burge, 2004).

The Gene Ontology (GO) enrichment analysis on microexon genes is calculated by the clusterprofiler (Yu et al., 2012) and visualized with an R language package.

## Results

### Identification of Microexons Using Multiple RNA-Seq Data by TopHat

**FIGURE 1:**
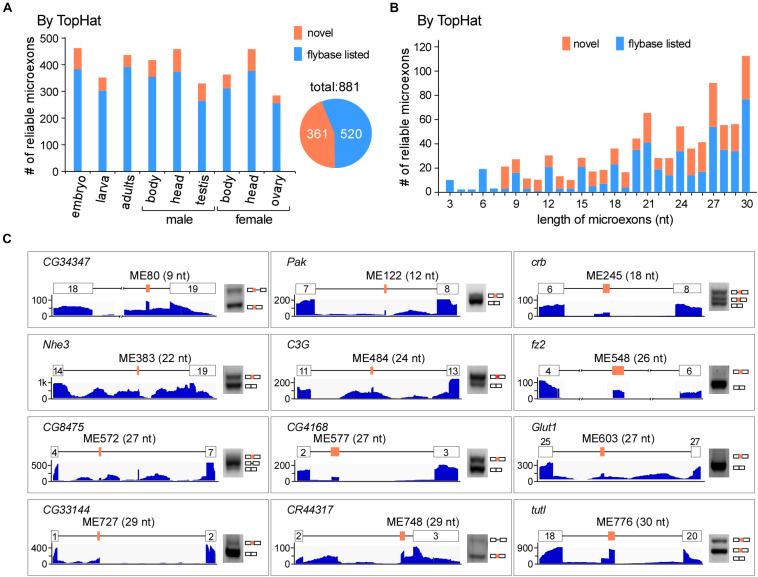
Identification of microexons in *Drosophila* by TopHat. **(A)** Identified reliable microexons from RNA-seq data of a variety of *Drosophila* samples by TopHat. Orange: novel microexons; blue: annotated microexons in Flybase. **(B)** Length distribution of the identified microexons by TopHat. **(C)** Validation of novel microexons by RT-PCR. Each microexon was validated by amplification of cDNA using primers located in its flanking exons. Lengths and numbering of microexons and the genes to which they belong are indicated. Blank boxes: flanking exons, orange boxes: microexons. Primers information is listed in Supplementary Table 1.

To comprehensively identify microexons in *Drosophila*, we collected a variety of our lab’s published and unpublished RNA-seq data of the WT fruit fly, including the head, body, and gonads from both females and males, as well as the embryo (18 h), 3L larva (wandering stage) and adults (2 h). In total, we obtained > 1.3 billion reads (∼430 G data) for subsequent analysis (Supplementary Table 2). Using the common alignment software TopHat and after performing the microexon identification and filtering process (Supplementary Figure 1), we identified 37,083 microexon candidates in *Drosophila*, of which 881 are highly reliable and 361 are novel microexons that were not annotated by the Flybase (Figure 1A). The length distribution of those reliable microexons, with and without novel ones, is obviously higher at the integral multiple of 3 nt than their nearby non-integrals (Figure 1B). To validate, we selected 12 novel microexons and performed RT-PCRs followed by individual Sanger sequencings; all of them showed proper bands and right sequences (Figure 1C), demonstrating that the above bioinformatic identifications are reliable.

### ce-TopHat, a Modified TopHat, Identifies More Microexons

**FIGURE 2:**
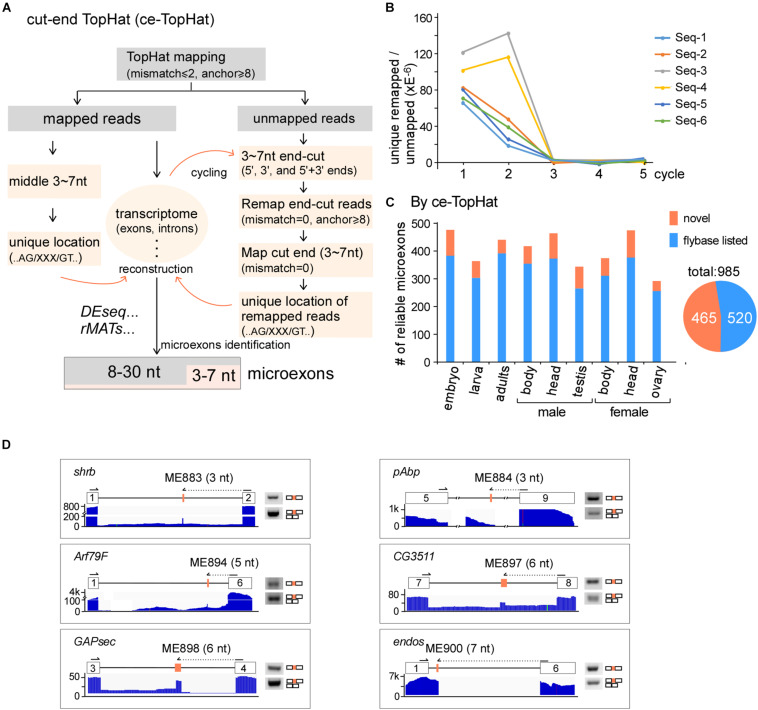
Identification of microexons in *Drosophila* by ce-TopHat. **(A)** Strategy for the identification of microexons by ce-TopHat. **(B)** Unique genomic locations of the remapped reads reach zero after three cycles of ce-TopHat. Six RNA-seq samples are indicated here, and other tested samples are shown in Supplementary Figure 2. **(C)** Identified reliable microexons from a variety of *Drosophila* samples using ce-TopHat. Orange: novel microexons; blue: annotated microexons on Flybase. **(D)** Validation of 3–7 nt novel microexons by RT-PCR. Arrows indicate the location of one set of PCR primers. All the PCR products were confirmed by Sanger sequencing.

Due to difficulties in mapping short segments to unique genomic locations, the above analyses did not find novel microexons that are shorter than 8 nt. To identify more microexons, especially in the lengths of 3–7 nts, we improved TopHat and developed a new computing model, named ce-TopHat (Figure 2A and Materials and Methods section). Briefly, ce-TopHat focused on mapping the 3–7 nt segments from the TopHat-unmapped reads to the *Drosophila* genome. There were two groups of unmapped reads with short segments according to their locations: (i) at the two ends and (ii) in the middle of the unmapped reads.

For the first group, each unmapped read could generate as many as 10 derivative reads by cutting 3, 4, 5, 6, and 7 nts at its 5′- and 3′-ends. Those cutting-end reads that could be remapped to the fly genome were collected, and sequences of short segments at their ends were used for searching locations in the nearby introns, sometimes in exons. We defined those short segments uniquely located in the genome and with flanking splice sites (AG/___/GT) as microexon candidates (Figure 2A right). After three rounds of remapping and transcriptome reconstruction, the recovered unmapped reads were decreased to near zero (Figure 2B and Supplementary Figures S2A,B), and in total, we remapped 7,369,109 reads from all the *Drosophila* RNA-seq samples (Supplementary Table 3). For the second group of unmapped reads, the 3–7 nt segments in the middle of remapped reads were also defined as microexon candidates if they had unique locations in the genome and flanking AG/___/GT sequences (Figure 2A left).

Remapped reads by ce-TopHat extended the fly transcriptome information and allowed us to identify an additional 104 highly reliable microexons (Figure 2C and Supplementary Table 4), of which 19 were 3–7 nts in length. The pattern of length distribution was not changed after adding those new microexons by ce-TopHat (Supplementary Figure 2C). Similarly, we then selected six microexons with a length of 3–7 nts for validation. Due to their short lengths, two sets of PCR primers were used for validation of each microexon. One set was primers both located in flanking exons; the other set contained one primer wholly located in one of the flanking exons and the other primer located in the other flanking exon with extended new microexon sequence. All of the six tested novel microexons were correct in RT-PCR bands and sequencing (Figure 2D).

Taken together, we identified 985 reliable microexons by ce-TopHat, of which 465 are novel microexons that were not annotated by the Flybase (Figure 2C).

### Microexons Are Enriched in Cell Morphogenesis and Neural-Related Genes

**FIGURE 3:**
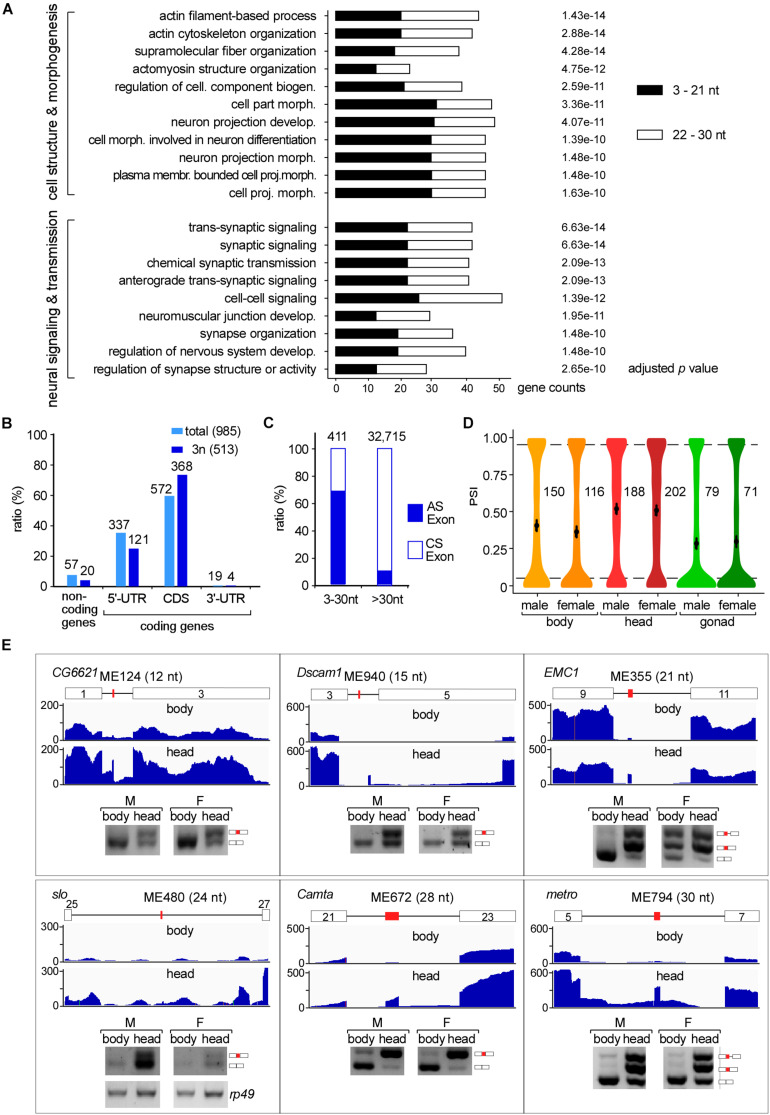
Tissues specific AS of Drosophila microexons. **(A)** Microexon-containing genes in *Drosophila* are highly enriched in two aspects of cellular functions by GO analysis. Enriched microexons are also divided into two groups based on length. **(B)** Distribution of microexons in gene regions. 3n: microexons in the length of integer multiples of 3. **(C)** Ratio of the AS exons is much higher in microexons than in longer exons. Microexons and longer exons that are located in the CDS region and have flanking exons were retrieved and analyzed. AS and CS exons were determined by transcriptomic information. **(D)** Distribution of the PSI values of AS microexons in *Drosophila* part/tissues. Average PSIs are indicated for each sample. **(E)** Validation of head-specific AS microexons by RT-PCR. Each microexon was validated by amplification of cDNA using two primers located in its flanking exons. Lengths, belonging genes, and names of microexons are indicated. Blank boxes: flanking exons, orange boxes: microexons. Primers information is listed in Supplementary Table 1.

We then performed GO analysis and found that microexon-containing genes in *Drosophila* are highly enriched in two aspects, the cell structure and morphogenesis (93 genes) and the neural-related signaling and development (80 genes), including pathways such as actin filament-based process and cytoskeleton organization, supramolecular fiber organization and cell part morphogenesis, neuron projection development and morphogenesis, synaptic signaling, neuromuscular junction development and synapse organization (Figure 3A).

To further characterize the microexon-containing genes, we split them into two groups based on the length of microexons, the 3–21 nt, and 22–30 nt groups. GO analyses revealed that the top 5 enrichments were different between these two groups. The 3–21 nt group was enriched in the aspect of cell morphogenesis, including cell part morphogenesis, cell morphogenesis involved in neuron differentiation, plasma membrane bounded cell projection morphogenesis, and neuron projection development and morphogenesis (Supplementary Figure 3A). However, genes in the 22–30 nt group were mostly enriched in the aspect of neural signaling and morphogenesis, including synaptic signaling, neuromuscular junction development, neuromuscular synaptic transmission, and synaptic signaling, as well as genes in the actin filament-based process (Supplementary Figure 3B). This observation implies that splicing would be different for various lengths of microexons, and this might be due to delicate splicing regulation in different environments. This is also consistent with previous studies, showing that alternatively spliced microexons play a major role in the development of the nervous system (Tsyba et al., 2004, 2008; Zibetti et al., 2010; Ohnishi et al., 2014; Toffolo et al., 2014).

### Alternative Splicing of Microexons

We further found that most *Drosophila* microexons were in coding genes, while only 5.8% were in non-coding genes. Among the coding genes, 58.1 and 34.2% of the microexons were in the CDS and 5′-UTR regions, respectively; few were found in the 3′-UTR region (Figure 3B light blue). Interestingly, microexons with the length of integer multiples of 3 nt (3n) exhibited significantly higher ratios (71.7%) in the CDS region (Figure 3B dark blue), suggesting that skipping or inclusion of most *Drosophila* microexons in CDS does not change much of their ORFs, but results in a difference in amino acids in a small number of proteins.

We then focused on the 411 exon-microexon-exon mode microexons, which have flanking exons on both sides (Supplementary Table 5). Among them, 68.9% (283) were alternatively spliced (AS) exons and 31.1% (128) were constitutive spliced (CS) exons. The ratio of AS in microexons is significantly higher than in the longer *Drosophila* exons, in which only 9.8% were alternatively spliced (Figure 3C). Analyses of the MAXENT scores, representing the strength of splice site signals, revealed that the strength of 5′SSs from AS microexons was at similar levels as their upstream 5′SSs, but the strength of 5′SSs from CS microexons was significantly higher than their upstream 5′SSs. A similar pattern was also found for the strength of 3′SSs (Supplementary Figure 4A). Consistent with this, consensus sequences of the upstream 5′SSs and downstream 3′SSs of CS microexons were less conserved than their counterparts of the AS microexons (Supplementary Figure 4B).

### Many AS Microexons Only Spliced in the Head

The alternative spliced 283 microexons were involved in 388 AS events (Supplementary Table 5). Percent-spliced-in (PSI) analysis revealed that AS events in the head from both females and males exhibited significantly higher average PSI values than in the bodies and gonads (Figure 3D), suggesting that a large portion of the AS microexons are actively spliced and included in transcripts in the *Drosophila* head. Comparison between the three parts of *Drosophila* using ΔPSI, an indicator for differential AS, also showed that the head was significantly different from the body and gonad in the splicing of microexons, while the difference between the bodies and gonads was much less (Supplementary Figure 4C). Interestingly, we found that 31 microexons were specifically spliced/included in the head and skipped in the bodies and gonads, while only four microexons were specific in the bodies and no microexons were specific in the gonads (Supplementary Table 6). These results strongly suggested that AS microexons have tissue specificities and splicing regulation of microexon is important for development and differentiation, especially in the head or the neuron systems. We also performed similar splicing analyses between our *Drosophila* developmental stage samples, including the embryo, larva, and adults. However, the results showed much less difference between the developmental stages than between the tissues.

To verify, we performed RT-PCR and analyzed splicing of differentially AS microexons between the heads and bodies, including the ME124 (12 nt in *CG6621*), ME940 (15 nt in *Dscam1*), ME335 (21 nt in *EMC1*), ME480 (24 nt in *slo*), ME672 (28 in *Camta*), and ME794 (30 nt in *metro*). The six tested microexons exhibited different AS splicing patterns between the two parts of *Drosophila*, in which more were spliced/included in the head samples (Figure 3E).

### Sexually AS Microexons in *Drosophila*

**FIGURE 4:**
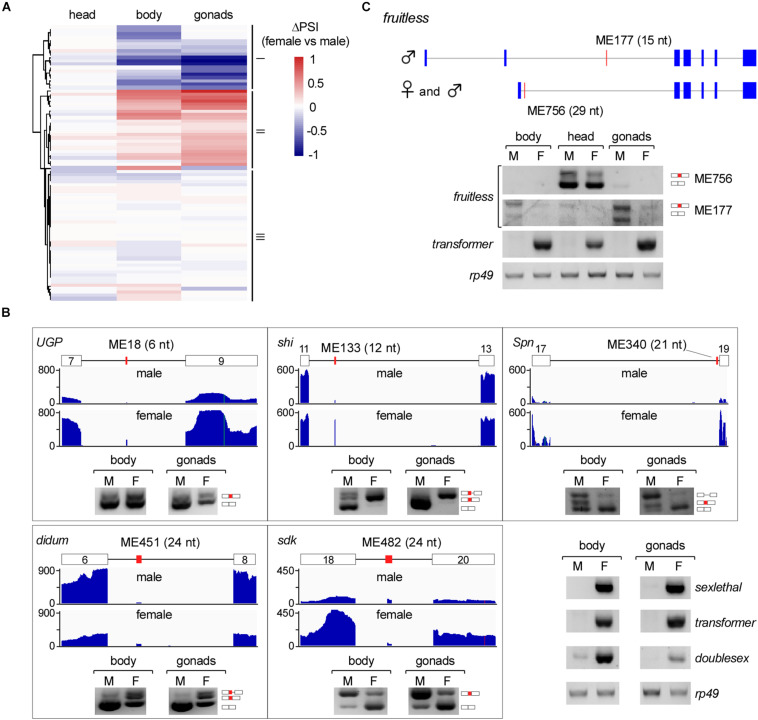
Sex-specific AS of *Drosophila* microexons. **(A)** Comparison of differential AS between samples from two *Drosophila* genders. **(B)** Validation of sexually AS microexons. Each microexon was validated by amplification of cDNA using two primers located in its flanking exons. Lengths, belonging genes, and names of microexons are indicated. Blank boxes: flanking exons, orange boxes: microexons. Primers information is listed in Supplementary Table 1. **(C)** Two microexons in *fruitless* are alternatively spliced.

We then analyzed differentially AS microexons in the three parts of *Drosophila* females and males. Unlike the above analyses, the female and male head samples showed few differentially AS microexons, while the sexually AS microexons mainly occurred in the bodies and gonads (Figure 4A). GO analyses showed that 61 genes containing the sexually AS microexons are mainly involved in the sensory system morphogenesis, cell structure organization, and the development of muscle cells, ovarian follicle cells, and epithelial cells (Supplementary Figure 5 and Supplementary Table 7).

To verify this, we tested five genes that contain sexually AS microexons, including the ME18 (6 nt in *UGP*), ME133 (12 nt in *shi*), ME340 (21 nt in *Spn*), ME451 (24 nt in *didum*), and ME482 (24 nt in *sdk*). Confirmed by RT-PCR, more ME18, ME133 and ME451 were spliced/included in transcripts in females than in males, and more ME340 and ME482 were spliced/included in males than in females (Figure 4B).

### The *fruitless* Gene Has Two AS Microexons

Among the genes in the sex determination pathway and sexual development, one of them, the *fruitless*, has two microexons, ME756 and ME177. ME756 (29 nt) is a novel head-specific microexon. Transcripts containing this microexon resulted in a premature termination codon and would decrease the protein level of Fruitless in the head. ME177 was previously annotated in the Flybase, here we found that it was specifically spliced in the male samples, especially in testis (Figure 4C).

## Discussion

Microexons are unique in both identification and function because of their short lengths. In this study, combined with deep sequencing of multiple *Drosophila* RNA samples and a new tool that recovers unmapped reads, we found hundreds of new microexons from the fruit fly transcriptomes and analyzed their differential AS between tissues and genders. These results demonstrate that more microexons could be identified through broader investigation of developmental and tissue/cell-specific transcriptomes, as well as the optimized and developed bioinformatic tools.

Most AS of microexons in previous studies were analyzed in the neural systems of mammals and fruit flies. In this study, we provide additional evidence that the AS of microexons are common in the *Drosophila* developmental stages, tissues, and gonads. These results imply that splicing regulation of microexons would be critical for the development of organs in *Drosophila*.

Lengths of exons and introns vary greatly in most species. The mode lengths of exons are 96 and 135 nt in humans and *Drosophila*, respectively (Supplementary Figure 6). Splicing of long-intron-flanking exons is usually considered in a mode of exon-definition in mammals, which defines exons by recognition of exonic splicing enhancers (ESE) as well as sequences of splice sites (Berget, 1995; De Conti et al., 2013). However, due to the short length, it has been speculated that the definition of microexons was also facilitated by sequences in the flanking introns (Scheckel and Darnell, 2015). Our identification of AS microexons in *Drosophila*, especially those 3–7 nts, would be useful to address such mechanisms in the future.

Another issue with microexons is their conservation across species. We performed evolutionary analyses of the *fruitless* ortholog genes and found that the sequence of the ME756 in *Drosophila* is highly conserved from zebrafish to human (Supplementary Figure 7). All of the sequences are located in the middle region for coding a BTB domain in the protein orthologs, suggesting an important function of this microexon in the fruit fly.

## Data Availability Statement

Next-generation sequencing has been submitted to the Gene Expression Omnibus (accession number GSE163147). All other data will be made available on request.

## Author Contributions

T-LP and Y-ZX designed the methodology. T-LP and ZD performed the bioinformatic analyses with help from S-BL. YZ, BZ, and LL carried out the investigation. T-LP and ZD performed the visualization. T-LP and Y-ZX wrote the original draft. Y-JF and Y-ZX acquired the funding and supervised the study. All authors contributed to the review and editing.

## Conflict of Interest

The authors declare that the research was conducted in the absence of any commercial or financial relationships that could be construed as a potential conflict of interest.

## Supplementary Material

Supplementary Figure 1 TopHat mapping, identification, and filtering of microexons.

Supplementary Figure 2 Remapping of the TopHat-unmapped reads by ce-TopHat.

Supplementary Figure 3 The top five GO enrichments of microexon-containing genes.

Supplementary Figure 4 Analyses of alternatively spliced microexons in *Drosophila*.

Supplementary Figure 5 Distribution and GO analysis of sexually AS microexons.

Supplementary Figure 6 Length distribution of exons in human, mouse, zebrafish, and fruit fly.

Supplementary Figure 7 The evolutionary analysis of BTB domain-containing orthologs of the *Drosophila fruitless* gene.

Supplementary Table 1 Primers used in this study.

Supplementary Table 2 Analyses of RNA-seq reads from fruit fly samples.

Supplementary Table 3 Remapping of unmapped-reads by ce-TopHat.

Supplementary Table 4 List of the identified reliable microexons in this study.

Supplementary Table 5 List of alternatively and constitutively spliced CDS microexons in *Drosophila.*

Supplementary Table 6 List of AS microexons that are specifically spliced and included in tissues.

Supplementary Table 7 List of significantly sexually AS microexons in *Drosophila.*
