# Supplementary material for: Comprehensive Identification and Alternative Splicing of Microexons in Drosophila
Source: Front Genet. 2021 Mar 30;12:642602. doi: 10.3389/fgene.2021.642602 (PMC8042270; doi:10.3389/fgene.2021.642602)
Supplement: Supplementary Figure 1 — TopHat mapping, identification, and filtering of microexons. [file Data_Sheet_1.PDF]

Supplementary Figures

Pang_Fig S1

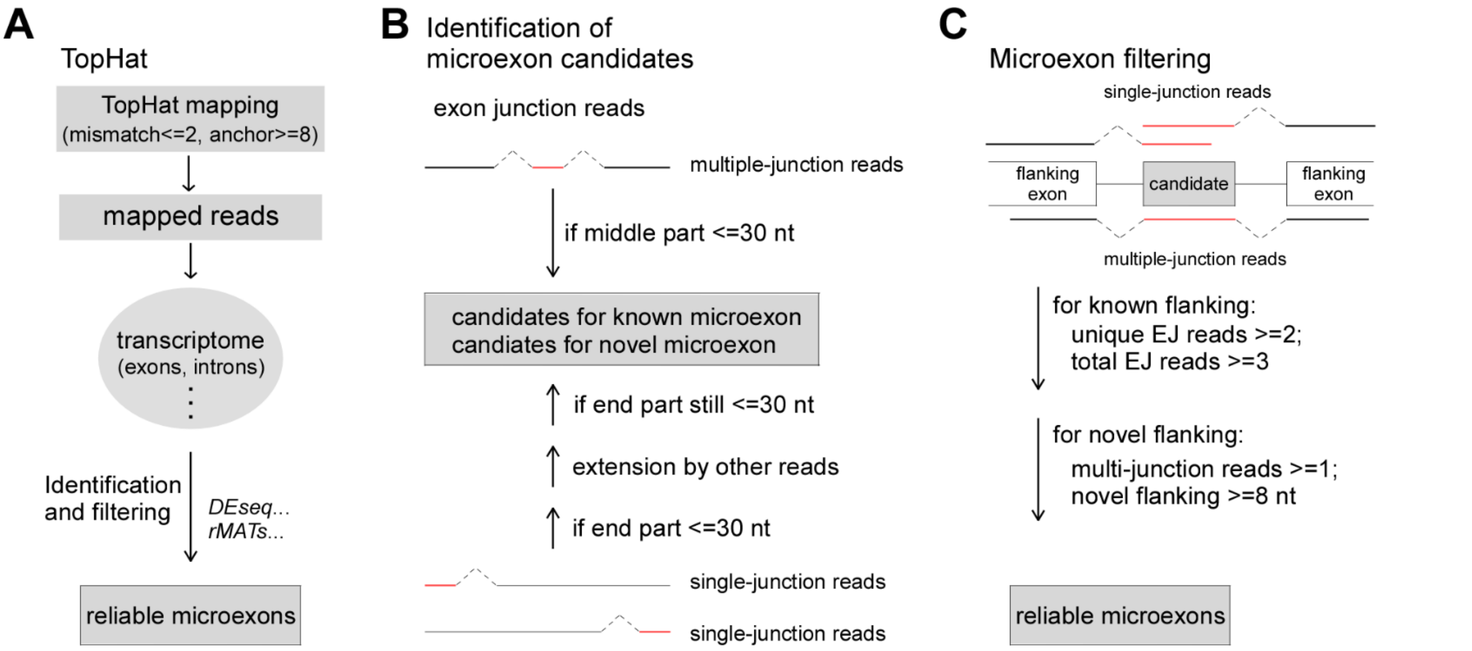

Figure S1. TopHat mapping, identification and filtering of microexons.
(A) The flow chart of TopHat mapping. The mapped reads generated by TopHat enrich the information of *Drosophila* transcriptome and is conducive to the downstream analysis. (B) The identification process of microexons in Drosophila transcriptome. (C) The filtering process to obtain highly reliable microexons.

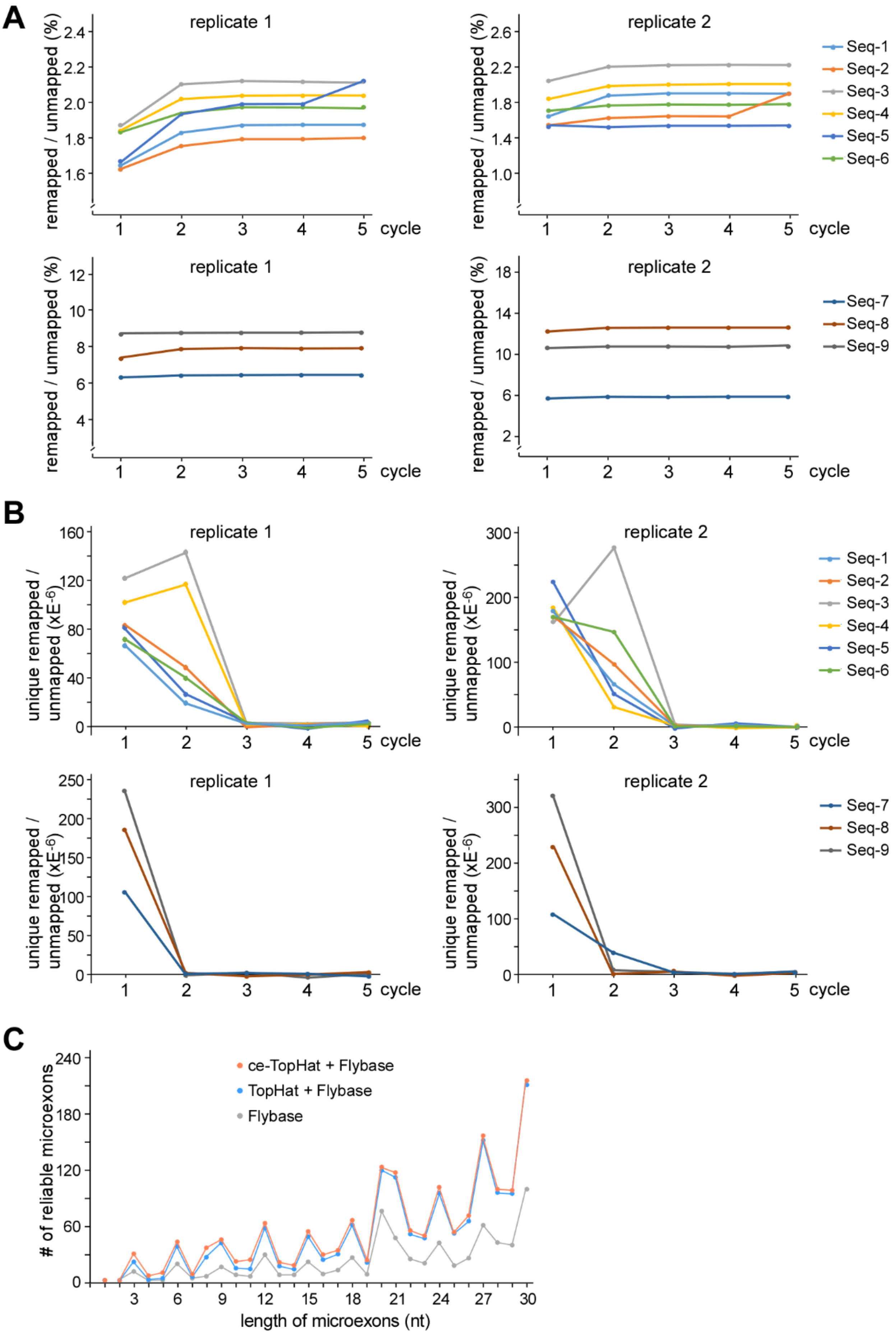

Figure S2. Remapping of the TopHat-unmapped reads by ce-TopHat.
(A) Ratio of the recovered reads during the cycles of ce-TopHat. (B) Ratio of uniquely remapped reads during the cycles of ce-TopHat. Applied sequencing samples and replicates are indicated, and their details are listed in Table S3. (C) The length distribution of microexons in *Drosophila*. Flybase listed, TopHat and ce-TopHat identified are compared.

Pang_Fig S3

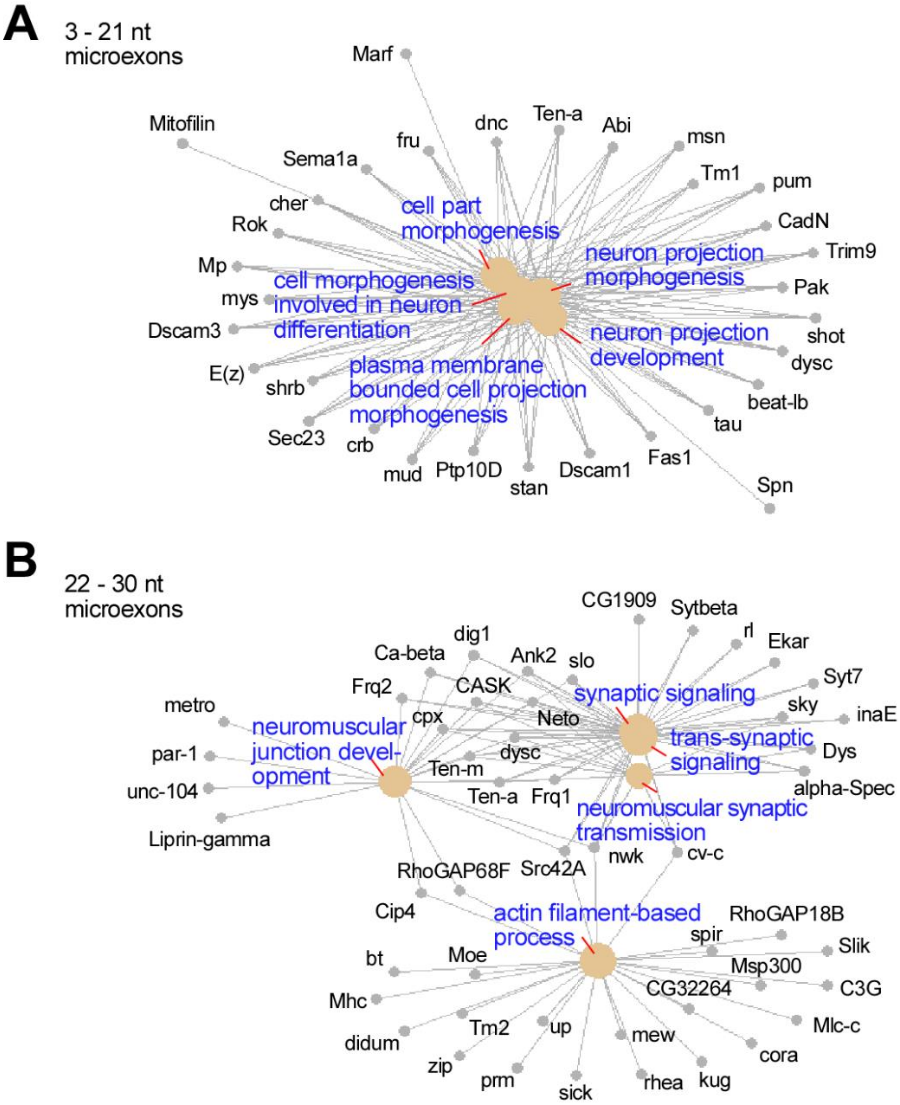

Figure S3. The top five GO enrichments of microexon-containing genes.
(A) The 3-21nt group were enriched in the aspect of cell morphogenesis. (B) The 22-30nt group were enriched in the aspect of neural signaling and morphogenesis.

Pang_Fig S4

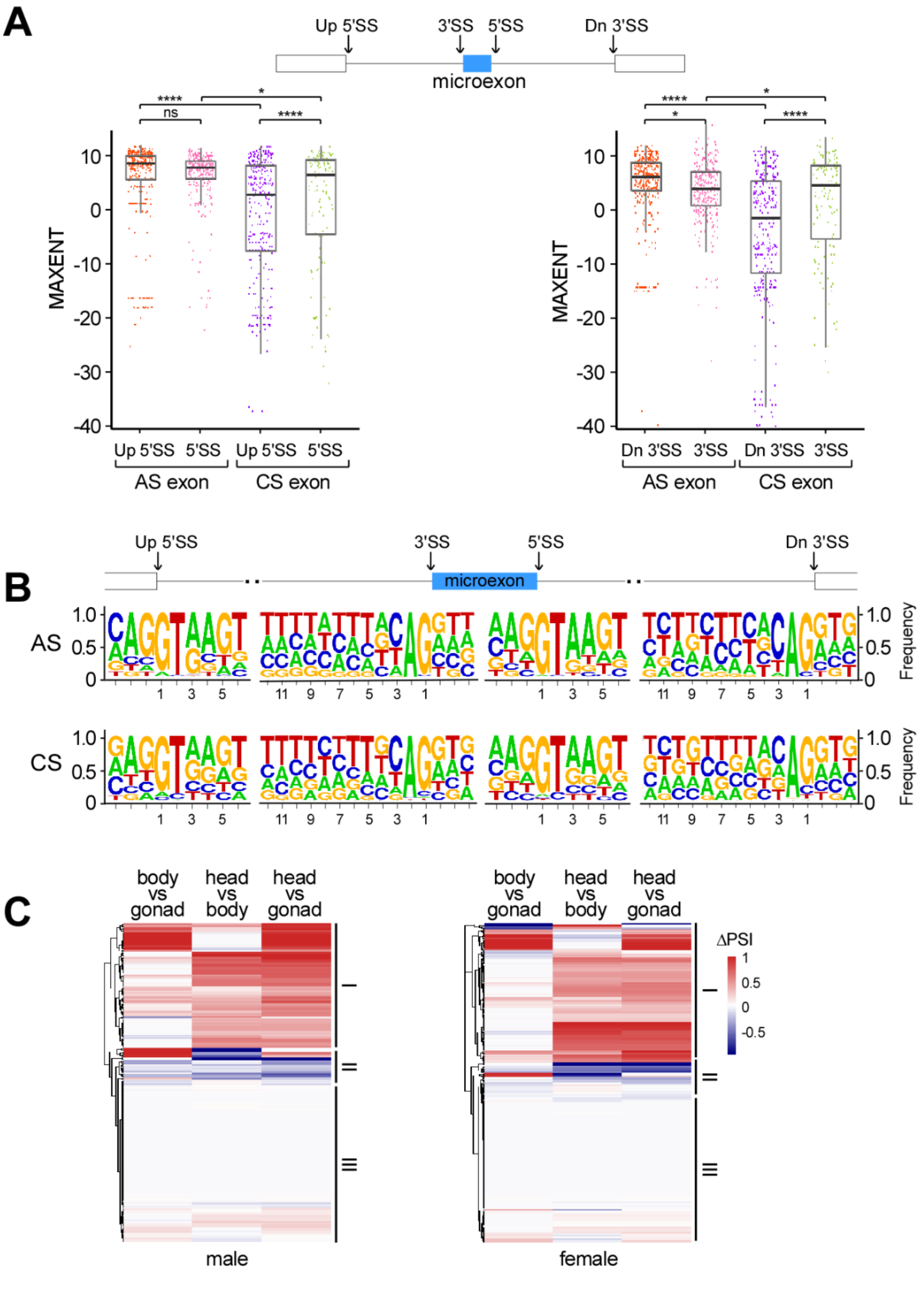

Figure S4. Analyses of alternatively spliced microexons in *Drosophila*.

(A) Flanking exons of AS microexons have stronger splice sites than those of CS microexons. Strength of the splice sites were calculated by online MaxEntScan. ns: P > 0.05; *: 0.01< P < 0.05; **: 0.001< P < 0.01; ***: 0.0001 < P < 0.001; ***: P < 0.0001.

(B) Consensus sequences of splice sites are more conserved in the AS microexons than in their flanking exons. Consensus sequences were obtained using Weblogo.

(C) Comparison of differentially AS microexons between Drosophila part/tissues.

Pang_Fig S5

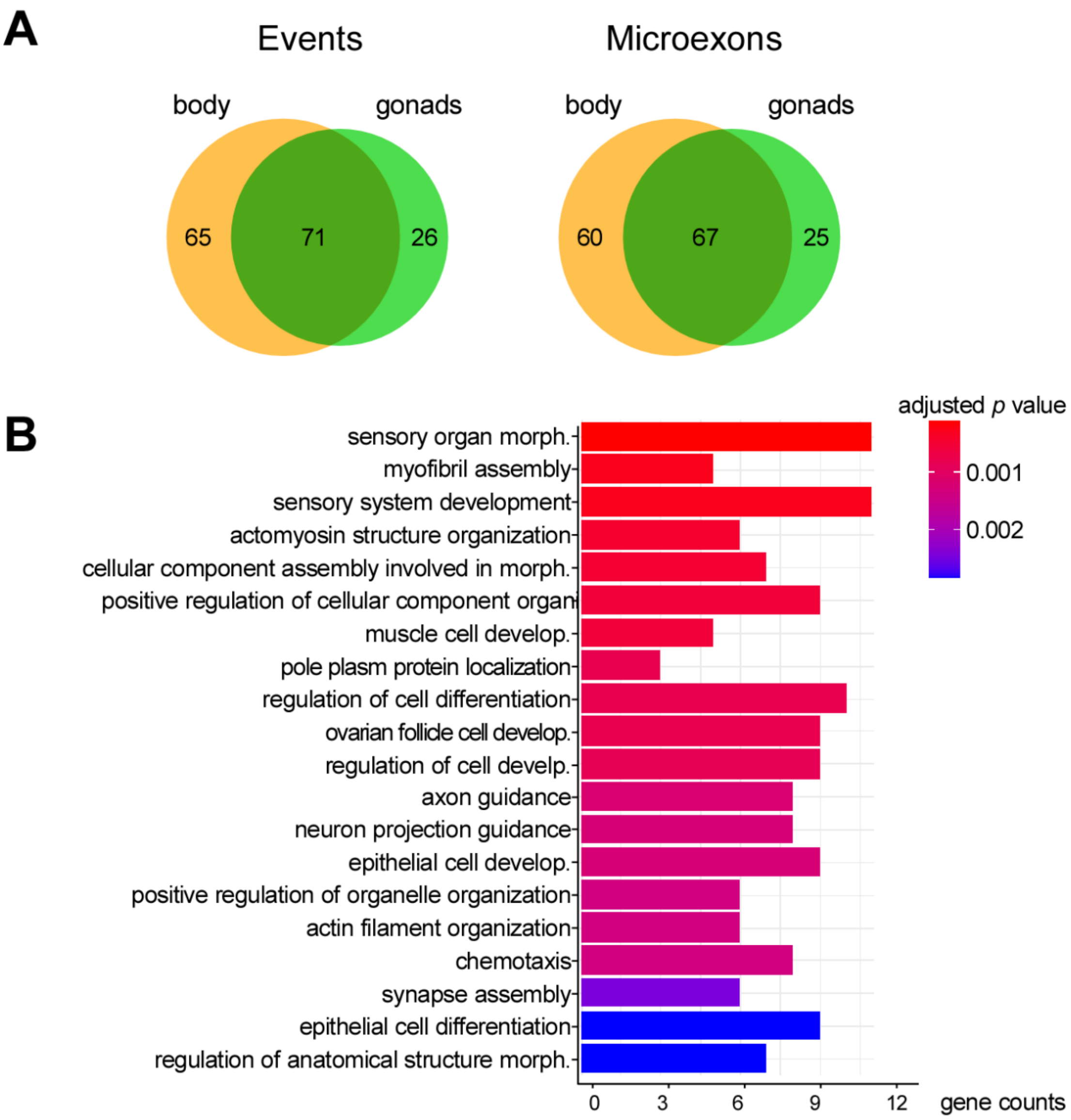

Figure S5. Distribution and GO analysis of sexually AS microexons.
(A) Distribution of significant sexually AS microexons between the bodies and gonads.
(B) GO analyses of significantly AS microexon-containing genes between the bodies and gonads from the females and males.

Pang_Fig S6

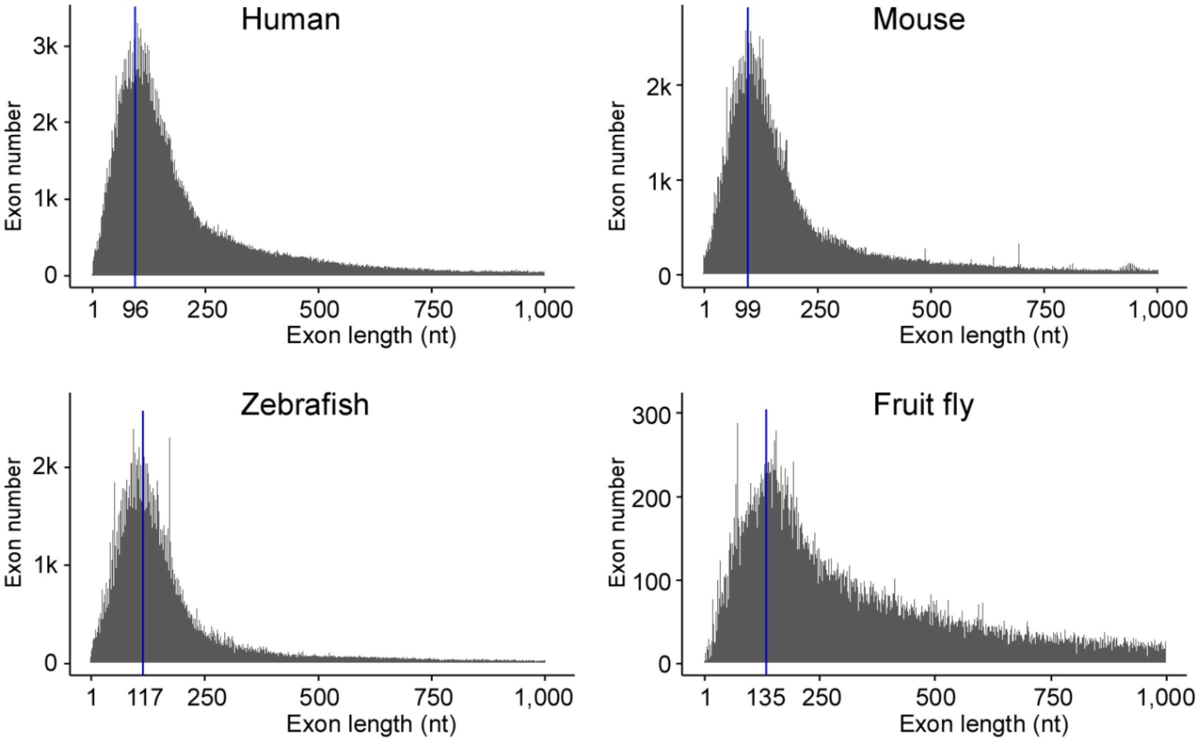

Figure S6. Length distribution of exons in human, mouse, zebrafish and fruit fly. The bule lines indicate the mode numbers of exon lengths.

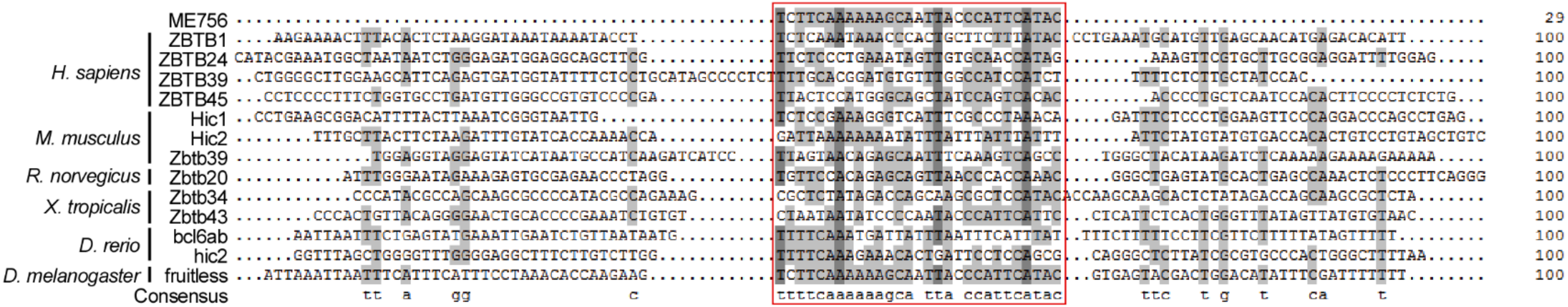

Figure S7. The evolutionary analysis of BTB domain-containing orthologs of the Drosophila *fruitless* gene. Evolutionary analysis of these orthologs indicates that the sequence of ME756 is highly conserved from zebrafish to human.
