# Supplementary material for: Comprehensive Identification and Alternative Splicing of Microexons in Drosophila
Source: Front Genet. 2021 Mar 30;12:642602. doi: 10.3389/fgene.2021.642602 (PMC8042270; doi:10.3389/fgene.2021.642602)
Supplement: Supplementary Table 2 — Analyses of RNA-seq reads from fruit fly samples. [file Table_2.DOCX]

**Pang _Table S2**

**Table S2. Analyses of RNA-seq reads from fruit fly samples**

| **Samples** | | **total reads** | **mapped reads** | **unmapped reads** | **Mapping ratio (%)** |
| --- | --- | --- | --- | --- | --- |
| embryo_1 | | 117,241,824 | 95,687,370 | 21,554,454 | 81.62 |
| embryo_2 | | 118,253,304 | 97,571,290 | 20,682,014 | 82.51 |
| larva_1 | | 67,487,514 | 60,711,727 | 6,775,787 | 89.96 |
| larva_2 | | 60,587,504 | 55,530,550 | 5,056,954 | 91.65 |
| adults | whole_adult_1 | 65,704,710 | 59,346,697 | 6,358,013 | 90.32 |
|  | whole_adult_2 | 60,478,566 | 55,800,584 | 4,677,982 | 92.27 |
|  | male_body_1 | 72,797,480 | 60,974,378 | 11,823,102 | 83.76 |
|  | male_body_2 | 61,983,426 | 51,944,251 | 10,039,175 | 83.80 |
|  | female_body_1 | 66,701,862 | 56,576,171 | 10,125,691 | 84.82 |
|  | female_body_2 | 74,151,982 | 62,744,481 | 11,407,501 | 84.62 |
|  | male_head_1 | 79,911,520 | 67,234,852 | 12,676,668 | 84.14 |
|  | male_head_2 | 53,995,634 | 44,282,059 | 9,713,575 | 82.01 |
|  | female_head_1 | 76,229,004 | 64,201,787 | 12,027,217 | 84.22 |
|  | female_head_2 | 71,752,986 | 59,897,675 | 11,855,311 | 83.48 |
|  | male_testis_1 | 69,695,962 | 57,748,828 | 11,947,134 | 82.86 |
|  | male_testis_2 | 60,619,146 | 49,902,835 | 10,716,311 | 82.32 |
|  | female_ovary_1 | 80,535,010 | 69,011,612 | 11,523,398 | 85.69 |
|  | female_ovary_2 | 66,575,160 | 57,161,026 | 9,414,134 | 85.86 |
