# Supplementary material for: Comprehensive Identification and Alternative Splicing of Microexons in Drosophila
Source: Front Genet. 2021 Mar 30;12:642602. doi: 10.3389/fgene.2021.642602 (PMC8042270; doi:10.3389/fgene.2021.642602)
Supplement: Supplementary Table 3 — Remapping of unmapped-reads by ce-TopHat. [file Table_3.DOCX]

**Pang _Table S3**

**Table S3. Remapping of unmapped-reads by ce-TopHat**

| **samples** | | **TopHat** | **ce-TopHat** | | | | | | **Notes** |
| --- | --- | --- | --- | --- | --- | --- | --- | --- | --- |
|  |  | **microexon candidates** | **cycle 1** | **cycle 2** | **cycle3** | **cycle 4** | **cycle 5** | **microexon candidates** |  |
| embryo_1 | | 2,714 | 1,365,150 | 1,388,523 | 1,389,515 | 1,390,711 | 1,390,910 | 4,180 | Seq-7 |
| embryo_2 | | 2,701 | 1,179,448 | 1,202,430 | 1,203,224 | 1,203,742 | 1,203,495 | 4,046 |  |
| larva_1 | | 2,366 | 499,278 | 533,897 | 537,207 | 535,807 | 537,219 | 3,592 | Seq-8 |
| larva_2 | | 2,418 | 617,177 | 635,047 | 636,138 | 635,453 | 635,405 | 3,498 |  |
| adults | whole_adult_1 | 3,009 | 551,890 | 557,610 | 557,706 | 557,705 | 557,659 | 4,359 | Seq-9 |
|  | whole_adult_2 | 2,900 | 495,925 | 501,638 | 501,717 | 501,792 | 501,645 | 4,203 |  |
|  | male_body_1 | 2,897 | 195,704 | 216,577 | 221,245 | 221,386 | 221,433 | 3,856 | Seq-1 |
|  | male_body_2 | 2,937 | 164,500 | 188,584 | 190,823 | 190,452 | 190,624 | 3,910 |  |
|  | female_body_1 | 2,192 | 165,402 | 178,200 | 182,312 | 182,345 | 182,325 | 2,922 | Seq-2 |
|  | female_body_2 | 2,426 | 175,753 | 184,659 | 187,038 | 187,036 | 187,036 | 3,336 |  |
|  | male_head_1 | 2,760 | 237,396 | 265,419 | 267,618 | 267,642 | 267,610 | 3,864 | Seq-3 |
|  | male_head_2 | 2,421 | 198,494 | 214,154 | 215,860 | 215,794 | 215,860 | 3,445 |  |
|  | female_head_1 | 2,695 | 221,386 | 242,522 | 244,750 | 244,836 | 244,717 | 3,629 | Seq-4 |
|  | female_head_2 | 2,603 | 218,272 | 234,886 | 237,546 | 237,582 | 237,508 | 3,767 |  |
|  | male_testis_1 | 2,965 | 200,273 | 231,272 | 237,422 | 237,435 | 237,405 | 3,919 | Seq-5 |
|  | male_testis_2 | 2,827 | 136,753 | 162,270 | 164,139 | 164,399 | 164,266 | 3,776 |  |
|  | female_ovary_1 | 2,117 | 211,299 | 222,840 | 226,931 | 227,022 | 226,965 | 2,758 | Seq-6 |
|  | female_ovary_2 | 2,139 | 160,244 | 166,146 | 167,046 | 167,037 | 167,027 | 2,854 |  |

**Notes:** After 5 cycles remapping by ce-TopHat, the total number of microexon candidates is 52,166, and the average per sample is 3,662.
